# Social support as a mediator between anxiety and quality of sleep among Chinese parents of special children

**DOI:** 10.3389/fpsyg.2023.1077596

**Published:** 2023-02-22

**Authors:** Junda Xu, Jiliang Tang

**Affiliations:** ^1^Zhejiang Rehabilitation Medical Center, Hangzhou, Zhejiang, China; ^2^School of Teacher Education, Taizhou University, Taizhou, Zhejiang, China

**Keywords:** anxiety, social support, sleep quality, coping style, Chinese parents of special children

## Abstract

**Objective:**

The psychological problems among Chinese parents of special children (mental retardation, limb disorder, hearing impairment, autism, cerebral palsy and other types) should be paid more attention. The aim of this study was to investigate the association between anxiety, social support, coping style and sleep quality among Chinese parents of special children during the early COVID-19 epidemic, so as to provide more help for the mental health of parents of special children scientifically and effectively.

**Method:**

A total of 305 Chinese parents of special children were invited to accomplish four questionnaires. Anxiety was measured by the Self-Rating Anxiety Scale, social support was evaluated by the Perceived Social Support Scale, sleep quality was assessed by the Pittsburgh Sleep Quality Index, and coping style was measured by the Simplified Coping Style Questionnaire.

**Results:**

This study revealed that anxiety was positively correlated with sleep quality (*p* < 0.01) and negatively correlated with social support (*p* < 0.01) and coping style (*p* < 0.01). Sleep quality was negatively correlated with social support (*p* < 0.01), but not significantly correlated with coping style (*p* > 0.05). Social support was positively correlated with coping style (*p* < 0.01). The study confirmed that social support had a partial mediating effect on the relationship between anxiety and sleep quality.

**Conclusion:**

The anxiety of parents of special children not only directly affects sleep quality, but also indirectly affects sleep quality through social support. Social support can alleviate the impact of anxiety on sleep quality through the mediating role.

## Introduction

Sleep quality which is always recognized as a very important physical and mental health index has been widely discussed (American Psychiatric Association, 2013). For example, studies found that lower sleep quality and sleep duration increase the risk of being overweight and obese (Parvaneh et al., 2016). Sleep disturbance is strongly associated with periodontitis, cardiovascular diseases and some cancers (Alqaderi et al., 2020). Lack of sleep leads to decreased work efficiency (Morgan, 1974), and poor sleep quality impairs emotion regulation and contributes depression symptoms (O'Leary et al., 2016). There are intertwined relationships discussed among sleep quality, physiological and psychological factors (Mohammadkhani et al., 2019; Taheri et al., 2019). Many studies have shown that parents of special children (mentally disabled children) have sleep problems. Parents of children with ASDs (autism spectrum disorders) reported poorer sleep quality compared to the TD (typically developing) group (Meltzer, 2008). Parents of children with developmental disabilities reported poorer sleep quality than parents of normal children (Gallagher et al., 2010). Children with neurodevelopmental disorders and their parents reported more severe sleep disturbances (sleep quality, insomnia symptoms and sleep efficiency) than typically developing children and their parents (Varma et al., 2021). Therefore, the quality of sleep has a big impact on body and mental health, and it is necessary to carry out further study for clarifying sleep quality related to mental health among parents of special children.

Which psychological factors affect sleep quality? Studies have shown that anxiety affects sleep quality. Most of studies have found a significant correlation between anxiety and sleep quality, and anxiety is a significant predictor of sleep quality (Augner, 2011; Liu et al., 2021; Simonetti et al., 2021; Yin et al., 2021). Many studies also have shown that social support is related to sleep quality (Brummett et al., 2006; Nordin et al., 2012; Guo et al., 2014; Marini et al., 2020; Mitchell et al., 2022). Some Studies have found positive coping style was negatively correlated with sleep quality (Kong et al., 2021) or not (Zhao et al., 2006), while negative coping style was positively correlated with sleep quality (Kong et al., 2021; Xue et al., 2012) or not (Zhou et al., 2013). In addition, studies have shown a correlation between anxiety and social support, coping style (Wu, 2010; Ma, 2013; Chen et al., 2016; Taş, 2019).

So, previous studies have found that anxiety, social support, coping style and sleep quality are related to each other. In addition, many studies have shown that the COVID-19 epidemic will affect individual sleep, anxiety and other mental health (Stanton et al., 2020; Qiu et al., 2021; Chatterjee, 2022). Studies have explored the psychometric relationship among these related variables, but the mediating role of social support and coping style between anxiety and sleep quality has been explored very rarely. How social support and coping style mediate the association between anxiety and sleep quality? Besides, selecting subjects of previous relevant studies mainly focus on college students.

Based on existing theories and researches, this study speculated that social support and coping style mediate the relationship between anxiety and sleep quality, and selected Chinese parents of special children as the psychological measurement subjects. The purpose of this study was to investigate the deep relationship among anxiety, social support, sleep quality and coping style of parents with special children, so as to provide some theoretical and practical proofs for the mental health of parents with special children during the COVID-19 epidemic period.

## Methods

### Participants

**Figure 1:**
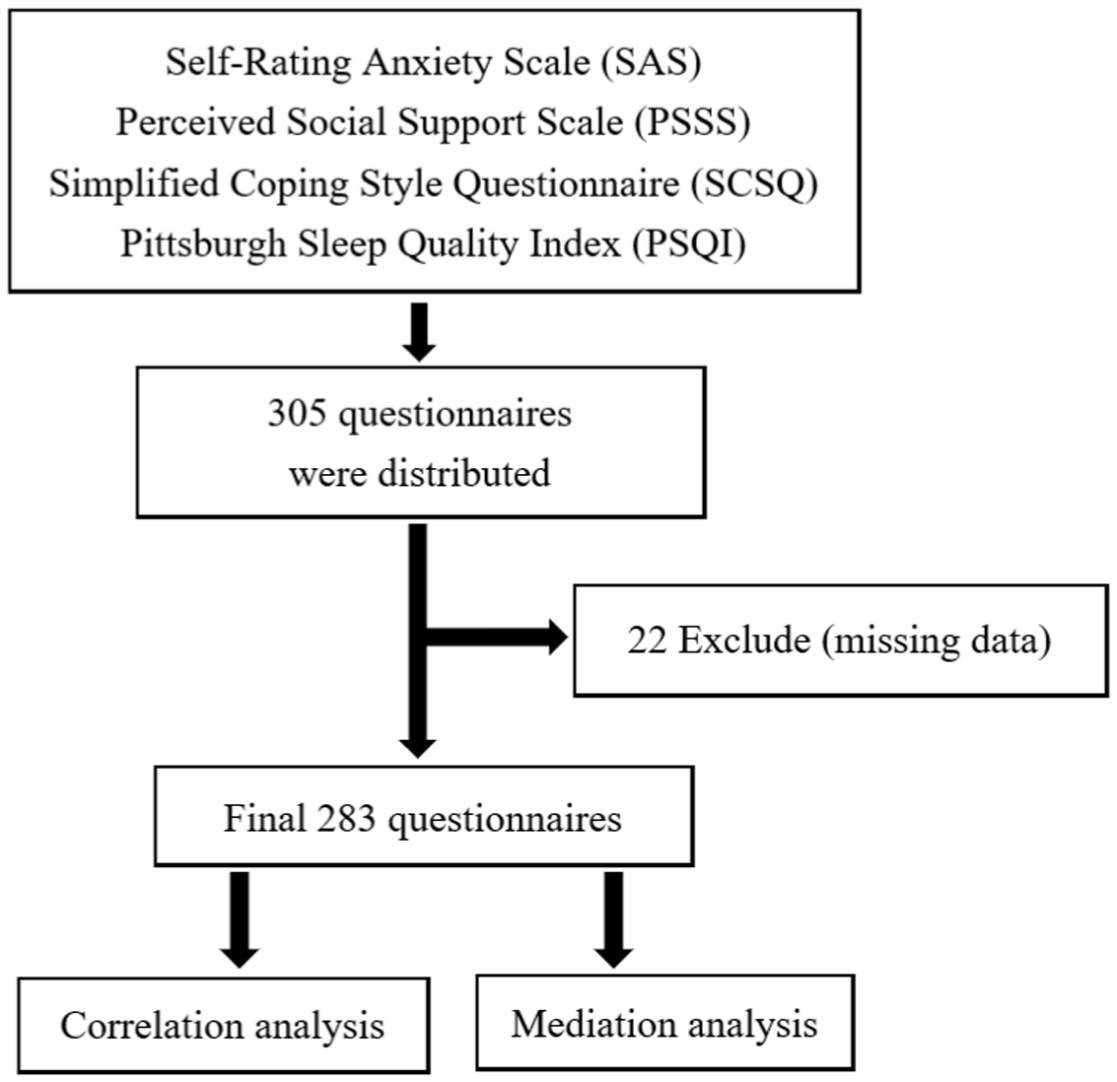
Study flow chart.

The Participants of this survey study were Chinese parents of special children refer to mental retardation, limb disorder, hearing impairment, autism, cerebral palsy and other types. This study conducted an online questionnaire to parents of special children in Hangzhou Green Apple Kindergarten in March 2020. A total of 305 questionnaires were distributed. After excluding missing data, a total of 283 questionnaires were included in this study. Two hundred eighty-three participants information see Table 1, and the study flow chart see Figure 1.

**Table 1:** Participants information.

Variables	Frequency (*n*)	Percentage (%)
Gender		
Female	231	81.6%
Male	52	18.4%
Education background		
Primary school	3	1.1%
Secondary school	49	17.3%
College students	216	76.3%
Graduate school	15	5.3%

## Measurement tools

### Self-rating anxiety scale

Anxiety was assessed with a 20-item Self-Rating Anxiety Scale by Zung (the scale derived from handbook: Wang et al., 1999). Ratings were made a 4-point Scale. Cronbach’s α of the scale in this study was 0.817. Confirmatory factor analysis indicated that the validity of this scale was good (χ^2^ =376.244; χ^2^/df = 2.650; *p* < 0.001; GFI=0.874; NFI=0.803; IFI=0.867; TLI = 0.817; CFI = 0.864; RMSEA = 0.076).

### Perceived social support scale

Social support was assessed with a Chinese revised version of Perceived Social Support Scale by Qianjin Jiang (the scale derived from handbook: Wang et al., 1999). The 12 items were rated on a 7-point Likert scale, ranging from 1 for strongly disagree to 7 for strongly agree with higher scores indicating greater social support. The Perceived Social Support Scale consists of three subscales: family support, friend support and significant others support. Cronbach’s α of the scale in this study was 0.932. Confirmatory factor analysis indicated that the validity of this scale was good (χ^2^ =122.376; χ^2^/df = 2.781; *p* < 0.001; GFI=0.935; NFI=0.949; IFI=0.966; TLI = 0.949; CFI = 0.966; RMSEA = 0.079).

### Simplified coping style questionnaire

Coping style in this study was assessed with a 20-item Simplified Coping Style Questionnaire developed by Yaning Xie (the scale derived from handbook: Wang et al., 1999). Ratings were made on a 4-point scale. The Simplified Coping Style Questionnaire consists of two subscales: positive and negative coping style. Cronbach’s α of the scale in this study was 0.875. Confirmatory factor analysis indicated that the validity of this scale was good (χ^2^ =396.742; χ^2^ /df = 2.681; *p* < 0.001; GFI=0.875; NFI=0.828; IFI=0.885; TLI = 0.849; CFI = 0.883; RMSEA = 0.077).

### Pittsburgh sleep quality index

Sleep quality was assessed with a Chinese revised version of Pittsburgh Sleep Quality Index by Xianchen Liu (the scale derived from handbook: Wang et al., 1999). This Scale had 24 items. The total score (without item 19 and 5 other evaluation items) ranged from 0 to 21, with higher scores indicating poorer sleep quality. Cronbach’s α of the scale in this study was 0.742. Confirmatory factor analysis indicated that the validity of this scale was good (χ^2^ =16.824; χ^2^/df = 2.103; *p* < 0.05; GFI=0.984; NFI=0.984; IFI=0.992; TLI = 0.978; CFI = 0.992; RMSEA = 0.063).

### Test of common method bias

Harman’s one-factor test was used to check Common Method Bias by exploratory factor analysis. As the results showed that characteristic values of 14 factors were greater than 1. The variation explained for the first factor of 14 factors was 19.015%, indicating that the variance interpretation of the maximum factor was below the upper limit standard of 40% (Podsakoff et al., 2003). Thus, there were no serious Common Method Bias of data in this study and further analysis can be used.

### Statistical method

Data statistics were implemented by SPSS (version 22.0). Applicable statistical methods were chosen to analyze the relationship among anxiety, social support, coping style and sleep quality in parents of special children.

## Results

### Descriptive analysis

Descriptive analysis results based on the questionnaire of 283 parents of special children are presented in Table 2. The average score of anxiety (SAS) was 32.10 (standard deviation: 6.92). For social support (PSSS), the average score was 57.61(standard deviation: 13.04). For sleep quality (PSQI), the average score was 4.84(standard deviation: 3.11). For coping style (SCSQ), the average score was 28.14(standard deviation: 9.68). Subscale average scores of the four variables are also presented in Table 2.

**Table 2:** Descriptive scores of variables.

Variables	*M* ± SD
Anxiety (SAS)	32.10 ± 6.92
Social support (PSSS)	57.61 ± 13.04
Family support	20.66 ± 5.02
Friend support	18.52 ± 4.94
Significant others support	18.44 ± 4.68
Coping style (SCSQ)	28.14 ± 9.68
Positive coping style	20.77 ± 7.66
Negative coping style	7.37 ± 3.90
Sleep quality (PSQI)	4.84 ± 3.11

### Correlation analysis

The results of correlation analysis (Pearson) are presented in Table 3. Sleep quality had significant positive correlation with anxiety (*p* < 0.01) and significant negative correlation with social support (included its three subscales “family, friend and significant others support”) (*p* < 0.01). There was nonsignificant correlation between sleep quality and coping style (included its subscale “positive coping style”) (*p* < 0.05), but the subscale “negative coping style” was positively correlated with sleep quality (*p* < 0.01). Anxiety had significant negative correlation with social support and coping style (*p* < 0.01). Social support and coping style had significant positive correlation (*p* < 0.01).

**Table 3:** Correlation analysis.

Variables	1	2	3	4	5	6	7	8	9
1. Anxiety (SAS)	1								
2. Social support (PSSS)	−0.343**	1							
3. Family support	−0.319**	0.856**	1						
4. Friend support	−0.330**	0.910**	0.639**	1					
5. Significant others support	−0.266**	0.906**	0.639**	0.795**	1				
6. Coping style (SCSQ)	−0.261**	0.298**	0.265**	0.237**	0.296**	1			
7. Positive coping style	−0.358**	0.356**	0.325**	0.302**	0.325**	0.925**	1		
8. Negative coping style	0.057	0.041	0.020	−0.005	0.097	0.665**	0.331**	1	
9. Sleep quality (PSQI)	0.470**	−0.297**	−0.285**	−0.320**	−0.183**	−0.026	−0.114	0.160**	1

*p < 0.05; **p < 0.01; ***p < 0.001.

## Mediation analysis

### Stepwise analysis method

**Figure 2:**
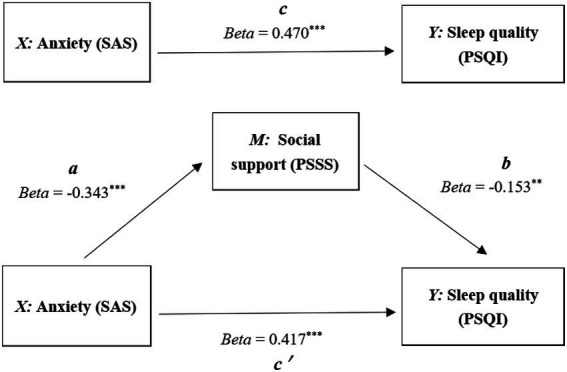
Mediating effect model.

The mediation effect was analyzed by using the stepwise analysis method (Baron and Kenny, 1986; Wen and Ye, 2014). In Figure 2, results indicated that anxiety played significant prediction on sleep quality (total effect coefficient “*c*”, *t* = 8.924, *p* < 0.001) and social support (indirect effect coefficient “*a*,” *t* = −6.128, *p* < 0.001); After bring into the mediation variable (social support), social support played significant prediction on sleep quality (indirect effect coefficient “*b*,” *t* = −2.769, *p* < 0.01), anxiety also played significant prediction on sleep quality (direct effect coefficient “*c*ʹ”, *t* = 7.529, *p* < 0.001). Therefore, social support partially mediated the relationship between anxiety and sleep quality.

Table 4 showed that in the stepwise regression equation, the direct effect (*c*ʹ) had allocated 88.72% explanatory effect, and the indirect effect (*a* × *b*) had allocated 11.17% explanatory effect. Obviously, social support as the mediating role leaded to this indirect effect producing.

**Table 4:** Effect analysis.

Effect style	Effect size	Explanatory percentage
Direct effect (*c*΄)	0.417	88.72% (Direct/Total)
Indirect effect (*a* × *b*)	−0.343 × −0.153 = 0.052479	11.17% (Indirect/Total)
Total effect (*c*)	0.470	

### Bootstrap analysis method

Beside the method of Stepwise, Bootstrap method (Hayes, 2013) was also made to test the mediating effect. According to the mediating effect test procedure proposed by Zhao et al. (2010), indirect effect “*a* × *b*” (confidence interval: LLCI = 0.0059, ULCI = 0.0503) was significant, and the direct effect “*c*ʹ,” (confidence interval: LLCI = 0.1382, ULCI = 0.2361) was also significant. So, bootstrap method suggested that social support partially mediated the relationship between anxiety and sleep quality as same as the stepwise method.

## Discussion

The aim of this study was to investigate the association between anxiety, social support, coping style and sleep quality among Chinese parents of special children during the early COVID-19 epidemic. The result found anxiety was significantly positively correlated with sleep quality, which was consistent with previous studies (Augner, 2011; Liu et al., 2021; Simonetti et al., 2021; Yin et al., 2021), and showed that social support also related to sleep quality closely, which was consistent with previous studies (Brummett et al., 2006; Nordin et al., 2012; Marini et al., 2020; Mitchell et al., 2022). This study found that coping style and its subscale “positive coping style” were not correlated with sleep quality, but the subscale “negative coping style” was positively correlated with sleep quality, which was partially consistent with previous studies (Zhao et al., 2006; Xue et al., 2012; Gao and Hu, 2021; Luo et al., 2022; Yu et al., 2022). So, we did not consider the coping style as the mediating role in the following analysis.

In this study, both stepwise and bootstrap analysis methods result suggested that social support partially mediated the relationship between anxiety and sleep quality. That means if parents of special children who have more anxiety will get worse sleep quality, while more social support can alleviate the impact of anxiety on sleep quality (Pittsburgh Sleep Quality Index with higher scores indicating poorer sleep quality) between anxiety and sleep quality. Like other studies with different subjects, the more social support was received or perceived, the better sleep quality was, and the social support could improve sleep quality (Guo et al., 2014; Wang et al., 2021; Xu et al., 2022). On the other hand, this study results suggest that the anxiety of parents of special children not only directly affects sleep quality, but also indirectly affects sleep quality through social support. The mediating role of social support can alleviate the impact of anxiety on sleep quality, and play a buffer role.

During early COVID-19 epidemic period (March 2020, also the time when parents answered the questionnaires), the Hangzhou Green Apple Kindergarten had delayed the start of school for a while. For parents of special children, they had to take care and train children at home themselves, without professional help of rehabilitation institute (the kindergarten) and without adequate outdoor physical activity. In this situation, as common mental health influence factors, anxiety and sleep problems may occur more obviously (Qiu et al., 2021; Blasco-Lafarga et al., 2022). We have seen that social support will be the key point for solving anxiety and sleep disorders through their logical relationship in this study. Therefore, increasing social support of parents of special children for reducing the impact of anxiety on their sleep quality is very necessary during COVID-19 epidemic with the prevention and control policy of school. Relevant organizations and charities should offer social psychological service and lead the society to give more understanding and support to these parents of special children.

## Conclusion

(1) Sleep quality had a significant positive correlation with anxiety and significant negative correlation with social support, but nonsignificant correlation with coping style; (2) Social support had a partial mediating effect on the relationship between anxiety and sleep quality. The anxiety of parents of special children not only directly affects sleep quality, but also indirectly affects sleep quality through social support. The mediating role of social support can alleviate the impact of anxiety on sleep quality through a buffer role.

## Data availability statement

The raw data supporting the conclusions of this article will be made available by the authors, without undue reservation.

## Ethics statement

The studies involving human participants were reviewed and approved by Ethics Committee of Zhejiang Rehabilitation Medical Center. All participants gave informed consent before be investigated.

## Author contributions

JT and JX designed the study. JX recruited the participants, distributed and collected the questionnaire. JX and JT analyzed the data, wrote the manuscript. JT and JX revised the manuscript. JT replied the office. JX submit for publication. All authors approved the final manuscript for publication.

## Funding

This work was supported by Taizhou Philosophy and Social Science Planning Project (22GHB24), and General Project of Zhejiang Provincial Education Department (Y201941753).

## Conflict of interest

The authors declare that the research was conducted in the absence of any commercial or financial relationships that could be construed as a potential conflict of interest.

## Publisher’s note

All claims expressed in this article are solely those of the authors and do not necessarily represent those of their affiliated organizations, or those of the publisher, the editors and the reviewers. Any product that may be evaluated in this article, or claim that may be made by its manufacturer, is not guaranteed or endorsed by the publisher.
